# Genome-Wide Analysis of the *Liriodendron chinense Hsf* Gene Family under Abiotic Stress and Characterization of the *LcHsfA2a* Gene

**DOI:** 10.3390/ijms25052733

**Published:** 2024-02-27

**Authors:** Yun Yang, Jianchao Yin, Liming Zhu, Lin Xu, Weihuang Wu, Ye Lu, Jinhui Chen, Jisen Shi, Zhaodong Hao

**Affiliations:** 1 State Key Laboratory of Tree Genetics and Breeding, Co-Innovation Center for Sustainable Forestry in Southern China, Nanjing Forestry University, Nanjing 210037, China; yunyang@njfu.edu.cn (Y.Y.); yinjianchao2023@163.com (J.Y.); zhulm@njfu.edu.cn (L.Z.); xulin@njfu.edu.cn (L.X.); wh879966@163.com (W.W.); luye@njfu.edu.cn (Y.L.); chenjh@njfu.edu.cn (J.C.); 2 Key Laboratory of Forest Genetics and Biotechnology of Ministry of Education, Nanjing Forestry University, Nanjing 210037, China

**Keywords:** *Liriodendron chinense*, *Hsf* gene family, *LcHsfA2*, abiotic stress

## Abstract

Heat shock factors (Hsfs) play a crucial role in plant defense processes. However, the distribution and functional characteristics of Hsf genes in the relict plant *Liriodendron chinense* are still unclear. In this study, a total of 19 *LcHsfs* were identified and divided into three separate subgroups, comprising 10 *LcHsfA*, 7 *LcHsfB*, and 2 *LcHsfC* genes, respectively, based on their phylogenetic tree and the presence/absence of conserved protein domains. Whole-genome duplication and segmental duplication led to an expansion of the *LhHsf* gene family. The promoters of *LcHsf* genes are enriched for different types of *cis*-acting elements, including hormone responsive and abiotic-stress-responsive elements. The expression of *LcHsfA3*, *LcHsfA4b*, *LcHsfA5*, *LcHsfB1b*, and *LcHsfB2b* increased significantly as a result of both cold and drought treatments. *LcHsfA2a*, *LcHsfA2b*, and *LcHsfA7* act as important genes whose expression levels correlate strongly with the expression of the *LcHsp70*, *LcHsp110*, and *LcAPX* genes under heat stress. In addition, we found that transiently transformed *35S:LcHsfA2a* seedlings showed significantly lower levels of hydrogen peroxide (H_2_O_2_) after heat stress and showed a stronger thermotolerance. This study sheds light on the possible functions of *LcHsf* genes under abiotic stress and identifies potentially useful genes to target for molecular breeding, in order to develop more stress-resistant varieties.

## 1. Introduction

Plants frequently encounter unfavorable growth conditions such as intense heat, cold, and drought during their life span. These stresses may severely limit the distribution of plants, alter their development, reduce productivity, and lead to male sterility [1,2]. In response to abiotic stress, plants have evolved multiple gene-regulatory mechanisms, involving stress sensing, signal transduction, transcriptional regulation, etc. Hsfs are at the core of the response systems, triggering the expression of heat shock proteins (Hsps) [3,4]. Therefore, it is essential to study *Hsf* genes in order to understand how plants respond to stress. 

The *Hsf* gene was first described in yeast in 1988 [5]. To date, *Hsf* gene homologues have been identified in the genome of most plant species, such as in *Physcomitrella patens* (8 genes), *Picea abies* (19), *Amborella trichopoda* (12) [6], *Oryza sativa* (25) [7], *Zea mays* (31) [8], *Brachypodium distachyon* (24) [9], *Vitis vinifera* (19) [10], *Arabidopsis thaliana* (21) [11], *Populus trichocarpa* (30) [12], *Malus domestica* (25) [13], *Capsicum annuum* (25) [14], *Fagopyrum tataricum* (29) [15], *Juglans regia* (29) [16], and *Camellia sinensis* (25) [17]. The aforementioned plants are classified as bryophytes, gymnosperma, basal angiosperms, monocots, and dicots, providing abundantly diverse resources for the study of *LcHsfs*. Typically, plant Hsf proteins consist of a DNA binding domain (DBD), an oligomerization domain (OD), and a nuclear localization signal domain (NLS). The Hsf family can be categorized into three subtypes, HsfA, HsfB, and HsfC, according to the characteristics of the OD [9]. Generally, HsfA proteins possess an activation domain (AHA), while HsfB proteins contain a repressor domain (RD) [18]. In *Arabidopsis*, HsfA have been shown to be transcriptional activators, while a number of HsfB transcription factors are likely to be transcriptional inhibitors [19].

*Hsfs* have been reported to play important roles in response to various stresses [20,21]. For example, the *AtHsfA1a*, *AtHsfA1b*, *AtHsfA1d*, and *AtHsfA1e* genes contribute to basal thermotolerance and also initiate the heat-stress response [22]. By contrast, cucumber (*Cucumis sativus*) *CsHsfA1d* knockdown lines are sensitive to cold stress [21]. Over-expression of *AtHsfA2* increases tolerance to heat, salt, osmotic stress, and high light intensity in transgenic *Arabidopsis* [23]. *AtHsfA3* expression is induced by a dehydration-responsive element-binding protein 2A (DREB2A) under heat stress [24]. Over-expression of *HsfA4a* increases salt tolerance and reduces H_2_O_2_ accumulation in transgenic plants [25]. Expression of *AtHsfA6a* is strongly induced by exogenous abscisic acid (ABA) [26]. The AtHsfA8 transcription factor (TF) is activated by H_2_O_2_ stimulation in protoplasts [27]. The TFs AtHsfB1 and AtHsfB2 repress the expression of heat-inducible Hsfs [19]. Hsfs have been well studied in model plants [12,17], but there have so far been few reports on their function in woody plants.

*Liriodendron* is an ancient relict plant, which belongs to the *Liriodendron* genus of the *Magnoliaceae* [28]. The *Liriodendron* genus consists of two distinct species: one East Asian species (*L. chinense* (Hemsley) Sargent) and one eastern North American (*L. tulipifera* Linn). A *Liriodendron* hybrid has been cultivated through the intraspecific hybridization between the aforementioned *L. chinense* and *L. tulipifera* [28]. It is used as a landscape tree species due to its unique leaf shape, gorgeous-looking flowers, and straight trunk. The *Liriodendron* hybrid was furthermore developed because of its excellent wood quality and fast growth [29]. However, as a perennial species, *Liriodendron* needs to adapt to seasonal variations and short-term environmental stresses. *Liriodendron* can tolerate mild drought and high temperatures. In sustained temperatures of 38 °C or higher, leaves may exhibit heat-damage symptoms, such as curling, yellowing, or even falling off. The *Liriodendron* genome sequence provides a foundation for molecular breeding strategies aiming to improve stress resistance [28]. In previous studies, the expression of *LcWYRK*, *LcCKX* (cytokinin oxidase/dehydrogenase), and *LcSOD* (superoxide dismutase) gene families were analyzed under various abiotic stresses [29,30,31]. However, a detailed study on the *LcHsf* genes family has not yet been reported and their transcriptional regulatory mechanism therefore remains to be elucidated. 

The aim of this study is to identify the *Hsf* gene family in the *Liriodendron* genome and to analyze the characteristics of these genes. Phylogeny, a conserved domain and motif analysis, as well as gene duplication and synteny analysis, were all performed. To elucidate the function of *LhHsf* genes, their expression patterns in different tissues and under abiotic stresses were determined. Using these data, we constructed an inter relatedness model between *LcHsf*, *LcHsp70*, and redox genes in response to heat stress. Further comparative analyses, such as promoter *cis*-element characterization, yielded a comprehensive understanding of the origin and evolution of the *Hsf* genes in *L. chinense*. This study provides insight into the function of *LcHsf* in abiotic stress and the important genetic resources for the formulation of stress resistance breeding strategies in the future.

## 2. Results

### 2.1. Identification and Chromosomal Location of the L. chinense Hsf Gene Family

**Figure 1:**
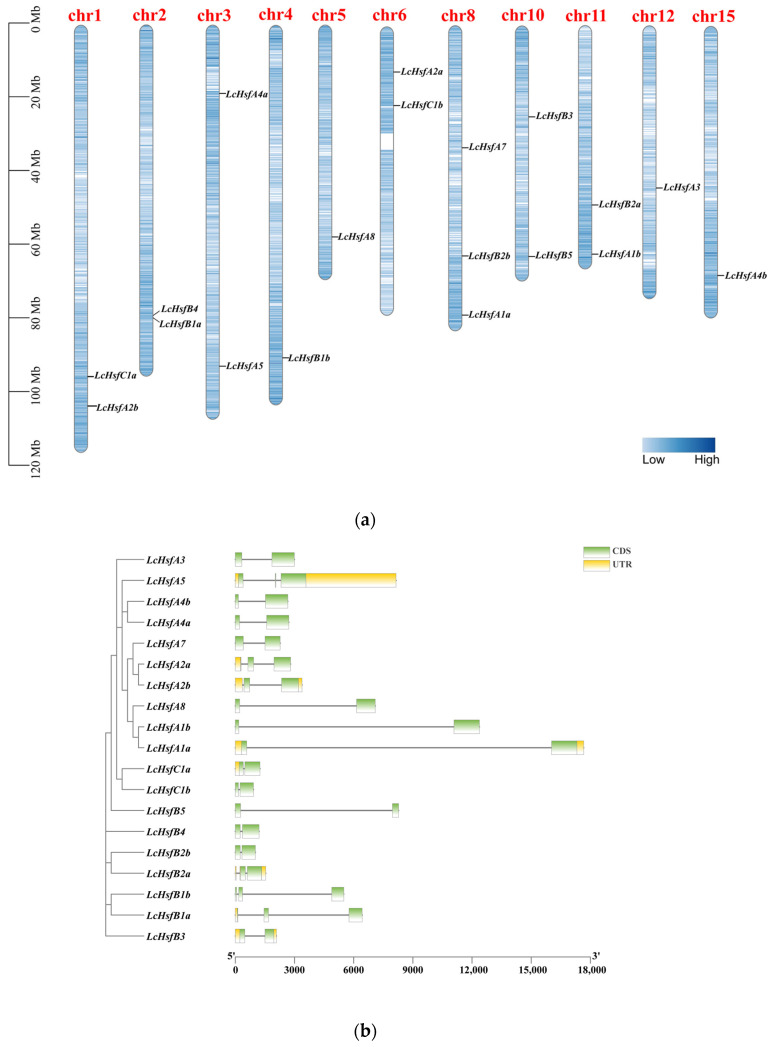
*LcHsf* gene characteristics. (**a**) The *LcHsfs* gene chromosomal loci allocated on 11 of 19 chromosomes. The scale is in million bases (Mb). The block-color-deepness on chromosome represented the different density of gene distribution, the deeper, the more genes distributed. (**b**) *LcHsf* gene structure. The green and yellow boxes represent the coding sequence (CDS) and the untranslated regions (UTR), respectively.

A total of 19 putative *LcHsf* family members were identified in the *L. chinense* genome and named according to their homologous *Hsfs* genes from *Arabidopsis*. We describe a number of basic characteristics of the LcHsf proteins in Table 1. Their amino acid lengths range between 193 and 514, with molecular weights (MWs) ranging from 22.36 kDa to 56.98 kDa, and theoretical isoelectric points (pIs) from 4.75 to 9.51. All LcHsfs are predicted to be localized in the nucleus. The 19 *LcHsf* gene locations are unevenly distributed across 11 chromosomes of *L. chinense* (Figure 1a). To further understand the *LcHsf* family, the gene structure of its members was analyzed (Figure 1b). The *LcHsf* gene size varies from 921 bp (*HsfC1b*, *Lchi08814*) to 17,660 bp (*HsfA1a*, *Lchi08322*). Their coding sequences (CDSs) are similar in length, but intron length varies greatly among the three groups. 

### 2.2. Phylogenetic Analysis of Hsf Proteins

**Figure 2:**
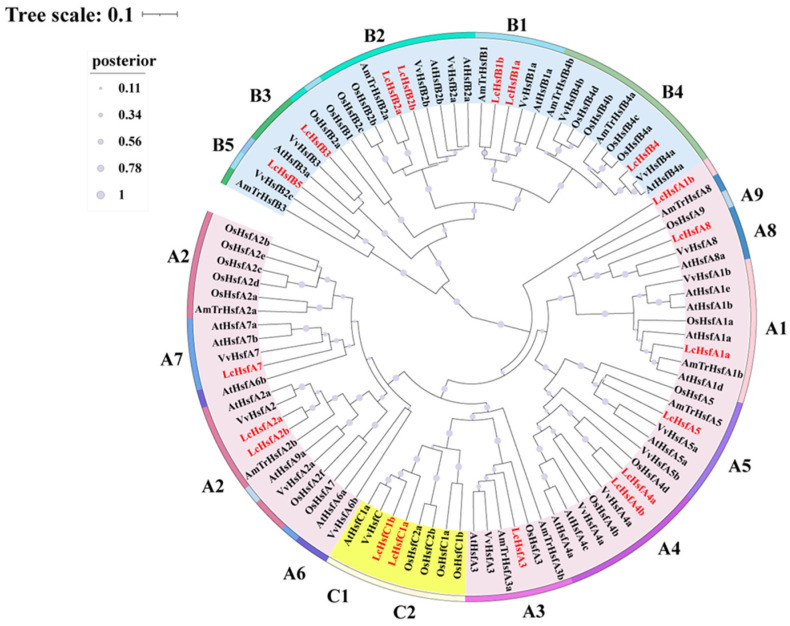
Phylogenetic tree of the Hsf family in *L. chinense* (Lc), *A. trichopoda* (AmTr), *O. sativa* (Os), *V. vinifera* (Vv), and *A. thaliana* (At). Pink represents the HsfA group, blue-green represents the HsfB group, and light yellow represents the HsfC group. Posterior values are displayed on the branches, represented by a lavender circle (see legend).

To evaluate the phylogenetic relationships within the Hsf family, we constructed a hierarchical cluster tree using the full-length protein sequences of five species, including *L. chinense* (Lc), *A. trichopoda* (AmTr), *O. sativa* (Os), *V. vinifera* (Vv,), and *A. thaliana* (At). All the Hsf proteins are divided into group A and group B, while group C and subgroup A3 cluster together (Figure 2). Among the 19 LcHsf members, 10 belong to the LcHsfA group and are relatively evenly distributed among subgroups A1–A8. There are seven members in the LcHsfB group and two members in the LcHsfC group. A total of 12 LcHsf proteins cluster in one group with AtHsf and VvHsf, while four LcHsf members are most closely related to their homologues from *A. trichopoda*: LcHsfA5, LcHsfB1b, LcHsfB2a, and LcHsfB5.

### 2.3. Conserved Domain and Protein Motif Analysis of LcHsf Subfamilies

**Figure 3:**
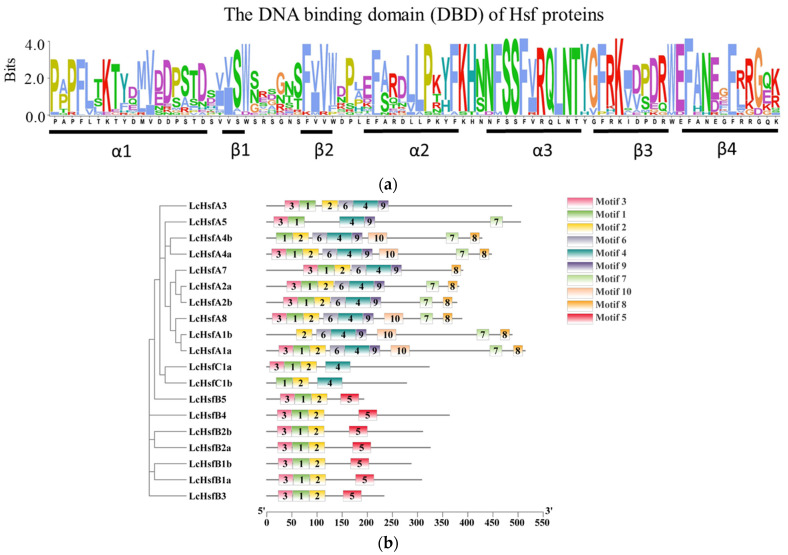
LhHsf protein DBD domain structure and protein motif distribution. (**a**) The LcHsf protein DBD domain is made up out of a α1-β1-β2-α2-α3-β3-β4 folding structure. A multiple sequence alignment analysis of the DBD domain of 19 predicted LhHsf sequences using ClustalX2.1 is shown. The bits score represents the sequence conservation score of all positions in the sequence. The higher this value, the better conserved it is. (**b**) A phylogenetic tree of all LcHsf proteins was constructed using the TBtool software (V2.001) and displayed on the left. The middle panel shows the result of a protein motif analysis via the Gene Structure View menu of TBtool (V2.001), with the 10 different motifs represented by a unique color scheme (see legend). Below is the x-axis, representing protein amino acid length, running from the N- (5′) to the C-terminus (3′).

All LcHsfs protein sequences contain a conserved DBD domain with an α1-β1-β2-α2-α3-β3-β4 structure, an OD domain, and an NLS domain (Figure 3a, Table 2). The LcHsf NLS domain has a typical sequence feature: NRR 5aa KKRR, RKRRLP, or KKRR. The nine LcHsfA proteins include an AHA domain, together with a nuclear export signal domain (NES). The LcHsfA AHA domains have characteristic amino acid residues (F, W), (I, L), and (E, D) at their C-terminus. For example, LcHsfA1a has an AHA domain with the sequence of “DSFWEQFLSA” (the underlines show the characteristic motifs of the domain and the following underlines are the same). The NES region of LcHsfA is rich in leucine; LcHsfB4, for example, has the typical NES sequence of “VLRKEDLGLNL” at its C-terminus. A hydrophobic, frequently leucine-rich NES at the C-terminus of Hsfs is required for the receptor-mediated nuclear export in complex with the NES receptor. Out of the six LcHsfB proteins, five possess an RD domain (the repressor structure -LFGV-), with the only exception of LcHsfB5. By contrast, the two LcHsfC members do not possess any domains in addition to the three domains shared by all LcHsf proteins (Table 2).

To better understand their protein structure, we searched for conserved motifs across all LcHsf proteins resulting in 10 individual identified protein motifs (Figure 3b). The majority of the LcHsf proteins contain motifs 1, 2, and 3 at their N-terminus. This can be attributed to the DBD domain being located near the N-terminus of all LcHsfs [9]. LcHsfA1b does not contain motifs 1 and 3, LcHsfA5 does not contain motif 2, while LcHsfC1b does not contain motif 3 due to the absence of several amino acids or the presence of a single nucleotide polymorphism site. Most LcHsfA proteins contain motifs 7, 8, and 9. On the contrary, LcHsfB proteins contain motif 5 at their C-terminus. As expected, more closely related proteins from the *L. chinense* phylogenetic tree have a similar motif composition.

### 2.4. LcHsf Gene Duplication and Synteny Analysis

**Figure 4:**
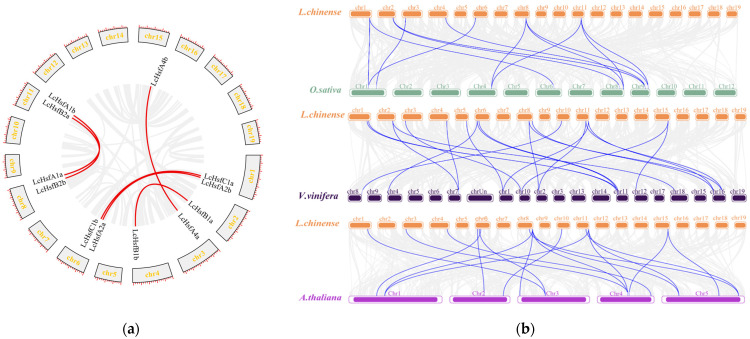
Genome-wide synteny analysis comparing the *L. chinense LcHsf* gene family to three other plant species. (**a**) Micro-synteny analysis of *L. chinense LcHsf* genes. The circle shows the 19 numbered chromosomes with red lines connecting homologous gene pairs. (**b**) Synteny analysis of *Hsf* genes between *L. chinense*, *O. sativa*, *V. vinifera*, and *A. thaliana*. The gray lines in the background indicate the collinear regions in the genomes of these four species, while the highlighted blue lines indicate the collinear *Hsf* gene pairs.

To determine the extent of expansion of *LcHsf* genes in *L. chinense*, we performed intra-species synteny analysis (Figure 4a) and found six gene pairs. Next, we searched for *LcHsf* gene duplication events. First, we could find no tandem repeat events within the *LcHsf* family. Second, according to MCscanX classification results, 12 out of the 19 *LcHsf* family members might have originated from whole-genome duplication (WGD) or segmental duplication events (Table S1). This finding suggests that the *LcHsf* family has expanded by WGD or segmental duplication. 

Since neofunctionalization of duplicated genes frequently is the result of natural selection pressure, we calculated Ka/Ks values (a non-synonymous/synonymous substitution ratio; Table 3). The Ka/Ks values of six *LcHsf* gene pairs were all less than 1. In addition, the Ka/Ks values of most of the *L. chinense* gene pairs are less than 1. The results are shown in Table S2 and Figure S1. To further explore the evolution of *LcHsf* gene families, we performed an interspecies synteny analysis of *L. chinense*, *O. sativa*, *V. vinifera*, and *A. thaliana*. These data show that there are 13 collinear gene pairs between *O. sativa* and *L. chinense*, 20 gene pairs between *L. chinense* and *V. vinifera*, and 18 gene pairs between *L. chinense* and *A. thaliana* (Figure 4b). These results indicate a close relationship of the *LcHsf* genes to their *V. vinifera* homologues. 

### 2.5. LcHsf Gene Promoter Cis-Element Analysis

**Figure 5:**
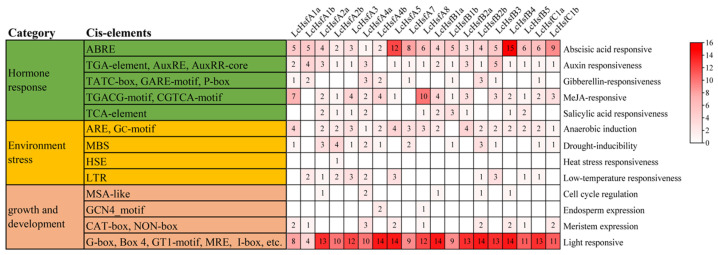
Statistics on the type and number of *cis*-elements in the promoter region of *LcHsfs* genes. Different types of *cis*-elements are classified on the left side of the graph, and the numbers in the heatmap indicate the numbers of different promoter elements.

*Cis*-regulatory elements in the promoter region provide specific transcriptional binding sites that affect gene expression. Therefore, we analyzed the presence of *cis*-acting elements. We found the *LcHsf* gene promoters to be enriched for various types of *cis*-acting elements (Figure 5 and Figure S4) and show a summary of all features in detail in the Supplementary Materials (Table S3). The first category of enriched elements is composed of hormone-responsive elements, including auxin responsive elements (TGA- and AuxRR-core elements), an abscisic acid responsive element (ABRE-element), methyl jasmonate (MeJA)-responsive elements (TGACG- and CGTCA-motifs), a salicylic acid responsive element (TCA-element), and gibberellin-responsive elements (a GARE-motif, TATC-box, and P-box). The second category is represented by abiotic stress response elements, including a heat stress responsive element (HSE-element), a drought-inducible element (MBS-element), a low-temperature-responsive element (LTR-element), a wound-responsive element (WUN-motif), and an anaerobically induced element (ARE-element). The third category is made up of plant-development-related elements, such as light-responsive elements (G-box, Box4, and GT), a meristem expression element (CAT-box), an endosperm expression element (GCN4_motif), and a seed-specific regulation element (RY-element). These results suggest that *LcHsf* genes might respond to different hormones and could be involved in various abiotic stresses responses as well as plant development.

### 2.6. Expression Profiling of Hsf Genes in Different Tissues of the Liriodendron Hybrid

**Figure 6:**
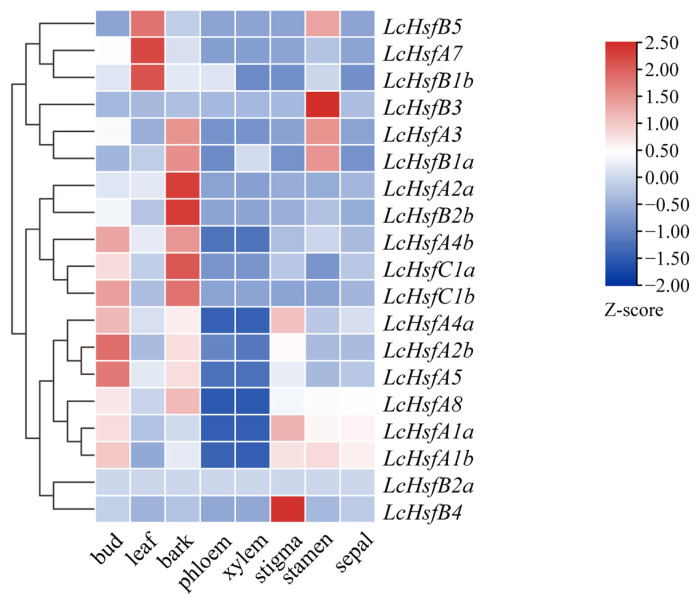
Expression profiles of *LcHsf* genes in eight different *Liriodendron* hybrid tissues: phloem, xylem, stigma, bark, bud, leaf, stamen, stigma, and sepal. The heatmaps were generated based on gene expression levels. The gene expression of fragments per kilobase million (FPKM) values were normalized using the TBtool software (V2.001) *Z*-score method, according to the following formula: $Z=\frac{x-\mu }{\sigma }$. μ refers to the data mean value, σ refers to the standard deviation value of the dataset and x refers to individual gene expression values. The legend bordering the heatmap shows the Z-value color code from −2.0 and +2.5. Genes with a high expression value are marked red, while those with a low expression value are marked blue.

To investigate the spatial expression patterns of *LcHsf* genes, their expression profiles were extracted from previously generated RNA-seq data. Most *LcHsf* genes were differentially expressed in vegetative and reproductive organs of *Liriodendron* (Figure 6). The *LcHsf* genes are actively expressed at their highest level on average in bud and bark tissue, followed by leaf and stigma tissue. By contrast, *LcHsf* genes are hardly expressed in the phloem and xylem. The *LcHsf genes* therefore demonstrate a tissue-specific expression pattern. *LcHsfB4* is highly expressed in the stigma and *LcHsfB3* is highly expressed in the stamen. These results suggest that the *LcHsf* genes are differentially regulated in different tissues. 

### 2.7. LcHsf Genes Show Diversified Expression Patterns under Heat, Cold, and Drought Stress

**Figure 7:**
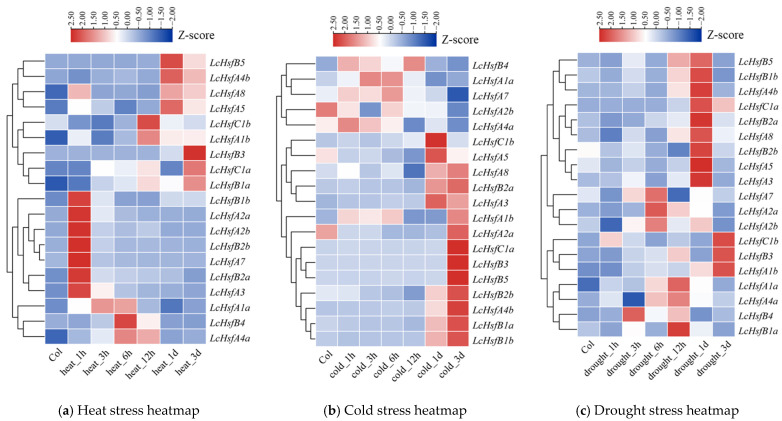
*LcHsf* gene expression patterns under heat (**a**), cold (**b**), and drought (**c**) stress for 1 h, 3 h, 6 h, 12 h, 1 d, and 3 d in *Liriodendron* leaves. The transcript abundance level was normalized and then hierarchically clustered. The gene FPKM values were normalized using the Z-score method. Blue blocks represent reduced transcriptional expression, while red blocks represent increased transcriptional expression.

To explore the possible role of *LcHsf* genes in response to various abiotic stresses, such as has been found in other plant species, *Medicago sativa* L. [32], *Panicum virgatum* L. [33], *Arachis hypogaea* L. [34], and *Populus trichocarpa* [12], we analyzed their expression patterns under heat, cold, and drought stress conditions (see Methods). We found that *LcHsf* A class (*LcHsfA2a*, *LcHsfA2b*, *LcHsfA3*, and *LcHsfA7*) and *LcHsf B* class (*LcHsf B1b*, *LcHsf B2a*, and *LcHsf B2b*) genes are highly expressed after one hour of heat treatment (Figure 7a). Supplementary Materials Figure S2 shows another heatmap of the *LcHsf* genes under heat stress, which was generated by the FPKM values directly. 

**Figure 8:**
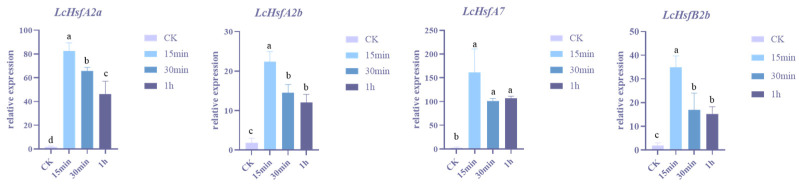
qRT-PCR analysis of selected high-responsive *LcHsf* genes in leaf tissues under heat stress. The relative mRNA abundance was normalized to reference gene of 18S rRNA. Gene expression values of a single gene denoted with non-identical letters are significantly different when assessed using a Duncan’s multiple range test (*p* < 0.05).

Previous studies in poplar and *Arabidopsis* found that *Hsf* gene expression can be detected after 15 or 30 min of heat stress [12,35]. We used quantitative reverse transcriptase PCR (qRT-PCR) to more closely follow the expression of *LcHsf* genes in *Liriodendron* seedlings leaf tissue (using the 166302 genotype) subjected to heat stress for various durations (Figure 8). We examined the expressions of four highly responsive genes (*LcHsfA2a*, *LcHsfA2b*, *LcHsfA7*, and *LcHsfB2b*) following heat stress treatment for 15 min, 30 min, and 1 h. The data indicate that all four *LcHsf* genes showed a significant increase in expression after 15 min of high-temperature stress and showed slowly decreasing but still elevated expression in the following 30 min and 1 h. Therefore, these *LcHsf* genes can be induced by heat stress in a short period of time. 

When testing the response to cold-stress treatment, we found three *LcHsf* genes (*LcHsfA3*, *LcHsfA5*, and *LcHsfC1b*) to be strongly induced after one day of treatment, while a further six *LcHsf* genes (*LcHsfA4b*, *LcHsfB1a*, *LcHsfB1b*, *LcHsfB3*, *LcHsfB5*, and *LcHsfB2b*) need three days to show strong upregulation (Figure 7b). 

Drought treatment induced the expression of different *LcHsf* family members at subsequent time points (Figure 7c). *LcHsfA2a*, *LcHsfA2b*, and *LcHsfA7* are upregulated after six hours of drought, while *LcHsfA1a* and *LhHsfB1a* show upregulation after twelve hours of drought. The majority of the *LcHsf* genes (*LcHsfA3*, *LcHsfA4b*, *LcHsfA5*, *LcHsfA8*, *LcHsfB1b*, *LcHsfB2a*, *LcHsfB2b*, and *LcHSfC1a*) require one day of drought treatment to show upregulation. Overall, these results show that most *LcHsf* genes respond vigorously to high-temperature stress at 40 °C, while their response to cold and drought stresses occurs in general at a slower pace. 

### 2.8. Transcriptional Correlation Networks between LcHsf, LcHsp70, and Redox Genes 

**Figure 9:**
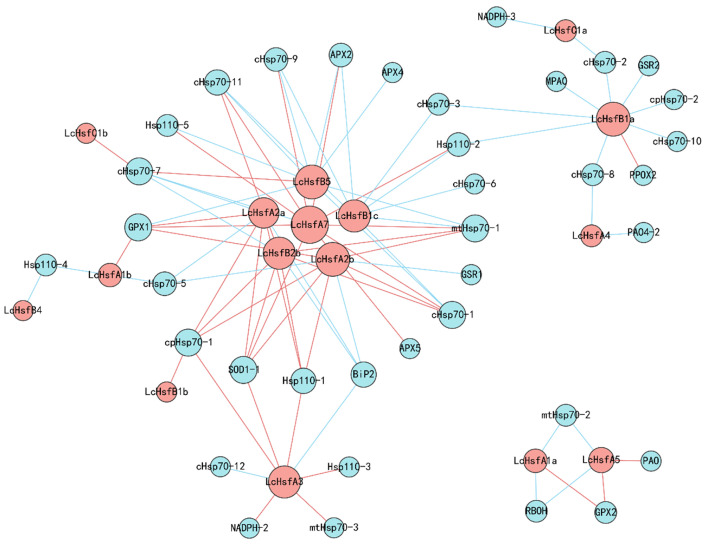
Transcriptional correlation network between *LcHsf* and *LcHsp70* genes. A connection represents a strong (r > 0.8) and significant (*p* < 0.05) correlation. The size of each circle is proportional to the number of connections. The higher the number of other points connecting a node, the larger the circle of the node. Red circles represent *LcHsf* genes while blue circles represent *LcHsp70* and redox-related genes. Red lines indicate that the expression levels of the two connected genes are positively correlated, while blue lines indicate that the two connected genes show negatively correlated expression levels. The *LcHsp70* superfamily includes cytosolic Hsp70s (*cHsp70*), ER-localized Hsp70s (*BiP*, Hsp70 homologs in the endoplasmic reticulum), mitochondrial Hsp70s (*mtHsc70*), chloroplast-localized Hsp70s *(cpHsc70*), and *Hsp110/SSE* family members. The redox-related genes include *RBOH* (respiratory burst oxidase), *PAO* (polyamine oxidase), *PPOX* (protoporphyrinogen/coproporphyrinogen III oxidase), *MPAO* (polyamine oxidase), *SOD* (superoxide dismutase), *NADPH* (monodehydroascorbate reductase), *GPX* (glutathione peroxidase), *APX* (L-ascorbate peroxidase), and *GSR* (glutathione reductase) genes.

Hsf TFs are responsible for the main heat-stress signal transduction network and regulate the expression of *Hsp70* genes [12]. Reactive oxygen species (ROS) act as signaling molecules that induce a heat-resistance mechanism and participate in the transduction network of Hsf/Hsp70 [4]. Therefore, it is useful to study a gene interaction model of Hsf, Hsp70, ROS, and ROS scavengers. We used the Pearson’s correlation method to evaluate the regulatory relationship among *LcHsf*, *LcHsp70*, and redox-related genes. We found that *LcHsfA2a*, *LcHsfA2b*, *LcHsfA7*, *LcHsfB1a*, *LcHsfB2bC*, and *LcHsfB5* act as the important genes of this network (Figure 9). *LcHsf* expression is highly positively correlated with the expression of *LcHsp70*, *LcHsp110*, *LcGPX*, *LcSOD1-1*, and the *LcAPX* gene family. Furthermore, the *LcHsp70* genes serve as nodes connecting *LcHsfA1b*, *LcHsfA3*, and *LcHsfB1a* to the core network. 

To better see the co-expression of *LcHsf*, *LcHsp70*, and redox genes, the weighted gene co-expression network analysis (WGCN) was carried out. The blue4 module of WGCN analysis contains the most *LcHsf* genes (Table S4). The blue4 module contains a total of 331,421 genes (Table S5). The edges of *LcHsf* genes with genes in the blue4 module are shown in Table S6. The result showed that, in the blue4 module, the *LcHsfB2b* gene had the most edges, 78 edges, with other genes, and there was a common edge with *SOD3*; *LcHsfA7* had the second most edges, 32 edges, with other genes, and there were no edges with LcHsp70 and redox-related genes; *LcHsfA2b* had 6 edges with other genes; *LcHsfC1* had 5 edges with other genes; and *LcHsfA2a* had 3 edges with other genes. *LcHsfA2b* has 6 edges with other genes; *LcHsfC1* has 5 edges with other genes; *LcHsfA2a* has 3 edges with other genes. The co-expression network is shown in Figure S3. These results would indicate that *LcHsfB2b* and *LcHsfA7* may play significant roles. Combined with the gene expression patterns and qRT-PCR results, the *LcHsfA2a* gene is also likely to be a factor in the regulation of heat stress.

### 2.9. LcHsfA2a Might Be Involved in Heat-Stress Response

**Figure 10:**
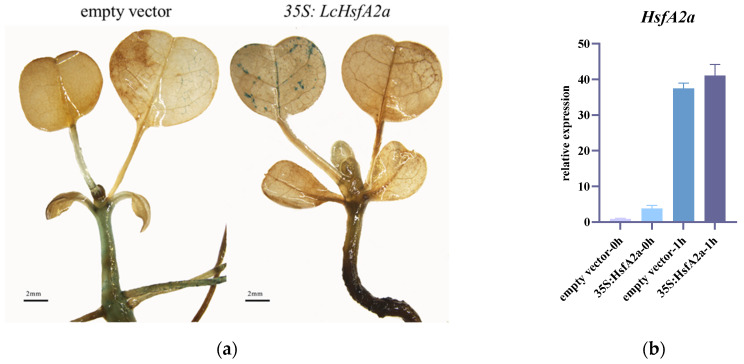
GUS staining and qRT-PCR analysis of *35S:LcHsfA2* transiently transformed plants. (**a**) Representative micrograph of GUS staining performed on empty vector and *35S:LcHsfA2a* transiently transformed plants. The scale bar is 2 mm. (**b**) Expression of *LcHsfA2a* in empty vector and 35S:*LcHsfA2a-iGUS* transiently transformed plants. The 0 h and 1 h refer to plants treated at 40 °C for 0 h and 1 h, respectively. Transgenic plants were transiently transformed for 48 h under normal conditions. The qPCR experiment uses technique replicates and biological replicates. The biological replicates form 3 separate transformation experiments.

Due to the important role of *HsfA2* in heat-stress response [14,36], we evaluated the possible functions of *LcHsfA2a* in thermotolerance. We amplified the coding region (CDS) of *LcHsfA2a* and cloned it following a 35S promoter (see Section 4.7). *Liriodendron* plants were transformed with both the empty vector *pBI121* and *35S:LcHsfA2a-iGUS* (β-glucuronidase gene, with introns). To identify transiently transformed plants, we performed a GUS staining and qRT-PCR analysis of *LcHsfA2a* gene expression. The plants transformed with an empty vector displayed GUS staining in both the stem and leaf, while *35S:LcHsfA2a-iGUS* transiently transformed plants exhibited stronger staining in the leaf (Figure 10a). At 48 h after transformation, plants growing under normal conditions were subjected to heat treatments for 0 h and 1 h, after which gene expression was analyzed. The expression level of *LcHsfA2a* in *35S:LcHsfA2a-iGUS* transiently transformed plants was three–four times that of empty-vector-transformed plants (Figure 10b). The expression of *LcHsfA2a* was increased after heat stress for 1 h in both control and *35S:LcHsfA2a-iGUS*.

**Figure 11:**
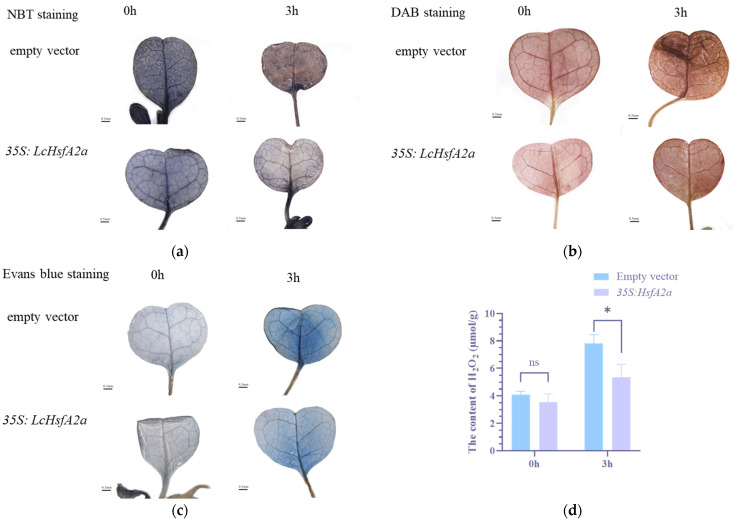
*LcHsfA2a* might be involved in heat-stress response. (**a**–**c**) DAB, NBT, and Evans blue staining of *35S:LcHsfA2a* and empty vector transiently transformed plants after 0 h or 3 h 40 °C heat stress treatment. NBT and DAB staining represent the accumulation of O^2−^ and H_2_O_2_ levels, respectively, while Evans blue staining reveals cellular death rate. The scale bar is 0.5 mm. (**d**) H_2_O_2_ content determination via colorimetric assay. The data were analyzed using a *t*-test (https://www.spsspro.com/, accessed on 29 August 2023). The * indicates the level of significance *p* < 0.05, ns means no significance.

To further understand the function of *LcHsfA2a* in heat-stress response, transiently transformed plants were treated with heat stress for 3 h; after which, nitrotetrazolium blue chloride (NBT), 3, 3′-Diaminobenzidine (DAB), and Evan’s Blue staining were performed to detect the content of O^2−^, H_2_O_2_, and cell damage. After a 3 h heat treatment, NBT staining showed that the leaves of *35S:LcHsfA2a* plants were light blue, while the leaves of empty-vector-transformed plants were damaged and appeared brown (Figure 11a). This shows that the superoxide radical (O^2−^) content after heat treatment is higher in *35S:LcHsfA2a* leaves, resulting in a healthier leaf overall. DAB staining showed the *35S:LcHsfA2a* leaves to have a lighter brownish color to the naked eye, in comparison to the empty vector leaves, which appeared a darker brown. This result indicated that the H_2_O_2_ level after heat treatment is lower in *35S:LcHsfA2a* leaves than in empty vector leaves (Figure 11b). 

An Evan’s blue staining found that both types of transiently transformed plants showed no apparent cell death before heat stress. After heat treatment for 3 h, cell death was reduced in leaves of *35S:LcHsfA2a* transiently transformed plants (Figure 11c). In addition, the H_2_O_2_ content in *35S:LcHsfA2a* transiently transformed plants was significantly lower after 3 h of heat stress (Figure 11d). This might be caused by overexpression of *LcHsfA2a* triggering the ROS scavenging system during the previous 48 h of transformation.

## 3. Discussion

The widely studied Hsf transcription factors are important regulators in heat-stress signal transduction pathways, and additionally play a role in the response to other abiotic stresses [37,38]. Until not, a genome-wide identification of the *Hsf* gene family in *Liriodendron* has not been published. Here, we report the classification, gene structure, promoter *cis*-acting elements and collinearity of *LhHsf* genes, as well as their expression patterns during abiotic stress response. In the current research, a total of 19 *LhHsf* genes were identified in the *Liriodendron* genome. *Liriodendron* has fewer *Hsf* gene members compared to previously studied monocot and dicot plants (Table S7). A recent study showed that the number of *Hsf* genes detected in higher plants was significantly higher than that in lower plants, based on the genome data of 103 higher plants and 8 lower plants [39]. For instance, the *Arabidopsis thaliana* genome possesses 21 *Hsf* genes. The *Arabidopsis* lineage underwent three rounds of WGD, two of which are assumed to have occurred approximately 60–70 and 23–43 Myr ago [40,41]. Since then, most gene duplicates have been lost. In addition, the soybean (*Glycine max* L.) genome contains 52 *Hsf* genes. The soybean lineage went through two rounds of WGD, which occurred approximately 59 and 13 Myr ago [42]. Compared to *Arabidopsis* and soybean, *Liriodendron chinense* experienced fewer and more ancient WGD events. An MCscanX classification indicated that 11 of the 19 *LcHsf* family members might originate from WGD or segmental duplication. It is generally believed that, after duplication events, some duplicated genes are removed from the genome due to random mutations and some are retained due to functional differentiation. This expansion of the *LcHsf* family has an adaptive effect on stress resistance in the process of continuous evolution.

*Cis*-acting regulatory elements are necessary for TF regulation and target gene expression. Our work shows that more than 90% of the *LcHsf* promoters contain abscisic acid responsive and auxin responsive elements, while nearly 85% contain Me-JA-responsive elements and over 50% contain gibberellin- and salicylic-acid-responsive elements. Furthermore, approximately 60% of the *LcHsf* promoters contain drought-inducible and low-temperature-responsive elements. These conclusions are similar to the findings of *cis*-acting regulatory elements on Hsfs family promoters in perennial ryegrass (*Lolium perenne*), common bean (*Phaseolus vulgaris*), and Tartary buckwheat (*Fagopyrum tataricum*) [15,43,44]. The ryegrass Hsf promoters also contain the same types of *cis*-elements, and five *LpHsfs* showed significantly differential expression patterns under various abiotic stresses [43]. *A. thaliana HsfA8*, *HsfB2a*, *HsfB2b*, and *HsfC1* are all responsive to abscisic acid treatment [23,25,45,46,47]. Previous studies have confirmed that their key *cis*-acting elements are highly related to abiotic stress response elements in plants [48]. Importantly, one article reported that DREB2A can induce *AtHsfA3* by binding to DRE elements in the *AtHsf3A* promoter under heat stress [24]. Supplementary Materials Figure S5 shows the TFs that bind to the Hsf promoter region from published reports. We are therefore intrigued whether the *LcHsf* promoter elements can similarly perform a corresponding response. Our results provide a basis for validating the function of *cis*-acting regulatory elements of *LhHsfs.*

In this study, *LhHsf* gene expression in response to high temperature, drought, and low temperature was analyzed using RNA-seq. During heat treatment, the expression of seven *LcHsf* genes (*LcHsfA2a*, *LcHsfA2b*, *LcHsfA3*, *LcHsfA7*, *LcHsfB1b*, *LcHsfB2a*, and *LcHsfB2b*) was significantly upregulated. The expression of these genes then gradually decreased after one hour, but these seven genes retained a continuous expression for at least three days. In comparison to this study, *HsfA2*, *HsfA3*, *HsfA7*, *HsfB1*, and *HsfB2* were stimulated by heat stress in *A. thaliana*, wheat (*Triticum aestivum* L.), maize (*Zea mays* L.), and tomato (*Solanum lycopersicum* L.) [36,49,50,51,52]. Concerning cold and drought stress, the major *LcHsf* genes (*LcHsfA4b*, *LcHsfA5*, *LcHsfA8*, *LcHsfB2a*, and *LcHsfB2b*) were upregulated on the first and third day. Similarly, several articles reported that *HsfA4a* can be induced by osmotic stress. *Malus domestica MdHSfA8* is activated under drought stress [53]. Interestingly, the heat-stress response (HSE-element), drought inducibility (MBS-element), and low-temperature response (LTR-element) were found in the *LcHsfs* promoters of genes responding to such stimuli. Of 19 *LcHsf* genes, 12 respond to a certain stress, and they also have corresponding element in their promoter (Table S8). These meaningful findings provide a reference for our research. And these results suggest that the majority of *LhHsf* genes might be involved in heat-, cold-, and drought-stress response regulation. 

The Hsf family is phylogenetically divided into three subfamilies: HsfA, HsfB, and HsfC. The LcHsf TF family contains 10 LcHsfA, 7 LcHsfB, and 2 LcHsfC homologues. It is reported that, in comparison to HsfB and HsfC, HsfA TFs play a more important role in responding to high temperature by rapidly activating thermal-dormancy-related gene expression, thereby helping plants to adapt to high-temperature stress [16]. In contrast, HsfB and HsfC TFs are more easily induced under low temperatures or other environmental constraints. This is in line with our research. Most *LcHsfA* genes are strongly expressed under heat coercion compared to *LcHsfB* and *LcHsfC* homologues. During the first hour of thermal coercion, the expression of *LcHsfA2a* increased by a maximum of 684 times, followed by an increase of 107 times of *LcHsfA7* and a 99-fold increase in *LcHsfA2b*. qRT-PCR results showed that the expression level of *LcHsfA2a* is significantly increased by 80-fold after 15 min of high-temperature stress, which is maintained for the following 1 h. 

In addition, plant HsfA homologues are involved in a network of protein–protein interactions, which add considerable complexity to their biological function under heat stress [54]. Network analysis based on RNA-seq data revealed that the expression of LcHsfA TFs is highly positively correlated with the expression of *cHsp70*, *LcHsp110*, *LcGPX*, *LcSOD1-1*, and *LcAPX* gene families. LcHsfA2a is the central regulatory factor under heat stress, as was shown in previously published research. We therefore selected it for further gene functional verification studies. The *35S:LcHsfA2a* transiently transformed seedlings displayed a H_2_O_2_ lower content after 3 h heat stress, implying that *LcHsfA2a* increases tolerance to heat stress in *Liriodendron*. In a previous study, it was shown that the expression abundance of *AtHsfA2* is more strongly induced by heat stress than the other *Hsf* homologues [23,55]. Overexpression of *AtHsfA2* resulted in significant accumulation of Hsp and ROS scavenging transcripts, such as galactinol synthase (*GOLS1* and *GOLS2*) and *APX1* [56]. These findings provided an excellent reference for analyzing the function of the *LcHsfA2* gene. *LcHsfA2a* might play a significant role in thermal adaptation and could be adopted for *Liriodendron* molecular breeding to improve such traits.

## 4. Materials and Methods

### 4.1. The Identification of the L. chinense Hsf Family

The gDNA, CDS, and protein sequences of *Liriodendron* were downloaded from the genome database (https://ftp.cngb.org/pub/CNSA/data1/CNP0000295/CNS0044063/CNA0002404/) (accessed on 26 September 2021). Candidate LcHsf protein sequences were searched via HMMER software (V3.0, Janelia Farm, Ashburn, VA, USA) using the Hsf Pfam number (PF00447). BLASTN similarity searches were performed with a threshold E-value of less than 1.0. In addition, all LcHsf proteins obtained were analyzed to detect specific protein domains using the conserved domains database (CDD) program (https://www.ncbi.nlm.nih.gov/cdd/) (accessed on 25 February 2022) [57]. The Hsf protein DBD domain “seqlogos” map was generated using Weblogo program of TBtool software (V2.001, South China Agricultural University, Guangzhou, China) [58]. The NLS domain amino acid sequence was detected using NLStradamus [59]. Basic protein properties, including protein length, molecular weight, isoelectric point, and molecular weight were determined using the ExPasy website (https://www.expasy.org/) (accessed on 25 February 2022) [60]. Subcellular localization was predicted via the WoLF PSORT program (https://wolfpsort.hgc.jp/) (accessed on 25 February 2022).

### 4.2. Phylogenetic Analysis and Identification of Gene Structures, Conserved Motifs, and Cis-Acting Elements

A phylogenetic tree was constructed using Hsf protein sequences of *Liriodendron chinense*, *Arabidopsis thaliana*, *Amborella trichopoda*, *Oryza sativa*, and *Vitis vinifera*. Their sequence IDs are listed (Table S9) and the sequences were downloaded from The *Arabidopsis* Information Resource (TAIR), Plant Transcription Factor Database (PlantTFDB V5.0) (https://planttfdb.gao-lab.org/) (accessed on 10 March 2022) , and Phytozome databases (v13, the Plant Comparative Genomics portal of the Department of Energy’s Joint Genome Institute, Washington, DC, America), respectively. Multiple sequence alignment was performed using ClustalX (V2.1, University College Dublin, Dublin, Ireland) software. Phylogenetic analysis was performed using BEAST (V2.6.4, University of Auckland, Auckland, New Zealand) with previously reported settings [30]. The iTOL website was used to visualize the phylogenetic tree (https://itol.embl.de/) (accessed on 14 January 2023) [61]. The exon–intron structure of *LcHsf* genes was analyzed by TBtool software (V2.001). Protein motifs were obtained from the MEME website (https://meme-suite.org/meme/) (accessed on 27 August 2022). The 2000 bp sequences upstream of the *LcHsf* transcription start sites were extracted by TBtool software (V2.001). *Cis*-acting elements were predicted using PlantCARE (https://bioinformatics.psb.ugent.be/webtools/plantcare/html/) (accessed on 4 March 2022) [62]. Analysis results were visualized using TBtool software (V2.001) [63].

### 4.3. LcHsf Gene Chromosomal Location, Gene Duplication, and Synteny Analysis

All *LcHsf* genes were mapped to their corresponding physical locations on a chromosome or scaffold. The chromosomal locations and circos plot resulting from *LcHsf* micro-synteny analysis were visualized by TBtool (V2.001). Gene duplication events were examined via MCScanX software (V1.0, University of Georgia, Athens, GA, USA) [64]. Collinear relationship maps of homologous *Hsf* genes were obtained for *L. chinense*, *O. sativa*, *V. vinifera*, and *A. thaliana* and displayed using the synteny visualization function of the TBtools platform (V2.001, South China Agricultural University, Guangzhou, China). Ka/Ks values were predicted by a Ka/Ks calculator (V3.0, National Genomics Data Center, Beijing, China) [65]. 

### 4.4. Determination of Tissue Specific LcHsf Gene Expression Patterns and Abiotic Stress Treatment of Liriodendron Hybrid Seedlings

All experimental materials were obtained from Nanjing Forestry University (Nanjing, China). Gene expression was quantified using RNA-seq data generated from different tissues, including bud, leaf, bark, phloem, xylem, stigma, stamen, and sepal (Table S10). Gene expression was examined after heat, cold, and drought stress (Table S11): 3-month-old seedlings were transferred to a growth incubator in preparation for stress treatment (23 °C, 16 h light and 8 h dark culture). To simulate heat stress, seedlings were treated with 40 °C for 1 h, 3 h, 6 h, 12 h, 1 day, and 3 days, respectively. To simulate cold stress, seedlings were treated with 4 °C for 1 h, 3 h, 6 h, 12 h, 1 day, and 3 days. And to simulate drought stress, seedlings were treated with 15% PEG 6000 for 1 h, 3 h, 6 h, 12 h, 1 day, and 3 days, respectively. Leaves were then selected for RNA-seq analysis. The datasets are available at NCBI (PRJNA679089 and PRJNA679101).

### 4.5. Construction of a Correlation Network Based on Gene Expression after Heat Stress

A Pearson’s correlation analysis was used to calculate an adjacency matrix between all *LcHsfs* from RNA-seq data generated under heat stress. The *LcHsf* gene expression file was set as one group, and the related *LcHsp70* and redox gene expression file was set as the second group. The *LcHsp70* superfamily included cytosolic Hsp70s (*cHsp70*), ER-localized Hsp70s (*BiP*), mitochondrial Hsp70s (*mtHsc70*), chloroplast-localized Hsp70s *(cpHsc70*), and *Hsp110/SSE* family members. The redox-related genes include *RBOH*, *PAO*, *PPOX*, *MPAO*, *SOD*, *NADPH*, *GPX*, *APX*, and *GSR* genes. Their expression file under heat stress is listed in Table S12. FPKM gene expression values were normalized to a computable log2 (value + 1 × 10^−6^). The correlation network of the two groups was calculated using the following settings: the filtering threshold of the association analysis was set to 0.8 and the *p*-value ≤ 0.05 [66]. The filtered correlation result is shown in Table S13. This work was performed using the Metware Cloud (https://cloud.metware.cn) (accessed on 28 August 2022). Graphical representation of the network was performed using Cytoscape [67]. 

The co-expression of *LcHsf*, *LcHsp70*, and redox genes was determined. All gene expression data under heat stress are shown in Table S14. Genes with the edge weight higher than 0.60 in blue4 module were selected for visualization (Table S15).

### 4.6. RNA Isolation and qRT-PCR

For short-term heat stress, qRT-PCR was used to quantify gene expression within 1 h after heat treatment. The *Liriodendron* hybrid seedlings were treated at 40 °C for 15 min, 30 min, or 1 h. The untreated control was cultured at 23 °C. Total RNA extraction from leaf tissues, cDNA synthesis, and qRT-PCR were performed according to previously published methods [30]. A 20 μL PCR reaction was set up. The *L. chinense* 18s gene was selected as internal reference. The primer sequences used are listed (Table S16). The data were analyzed using a 2^−ΔΔCT^ method. The raw qPCR data of four genes (*LcHsfA2a*, *LcHsfA2b*, *LcHsfA7*, and *LcHsfB2b*) are provided in Table S17.

### 4.7. Cloning of LcHsfA2a and Transient Transformation

To overexpress *LcHsfA2a*, its 1149 bp CDS was amplified from *Liriodendron* hybrid cDNA using specific primers (primer F: ATGAAATTTCACTTGCAGGAGAAGGC, and primer R: TCATGACTTTGACCCCAGAAAAC). The basic vector used was pBI121 without modification, which results in detectable GUS staining when transformed into *Liriodendron* seedlings. To ensure the accuracy of the transient transformation experiment, the iGUS gene (with introns) was cloned into pBI121 to replace the present GUS gene. The iGUS gene was cloned from the plant expression vector pcambia1301. The overexpression vector *35S:LcHsfA2a-iGUS* was subsequently generated by Gibson Assembly. Transformation of *Liriodendron* hybrid plants was performed according to a previously published article [68]. In brief, plant seedlings were immersed in transformation solution (1.0 OD Agrobacterium tumefaciens, pH 5.6) while swaying in the dark at 25 °C, 95 rpm for 4 h. Seedlings were then cultured on 3/4 MS medium for 72 h before GUS staining. Transient transformation experiments were repeated 3 times. GUS staining was performed according to a method previously described [69]. Briefly, seedlings were soaked in pre-chilled 90% acetone for 20 min, then the seedlings were washed with X-Gluc-free staining solution after the acetone was removed. Next, the seedlings were evacuated by vacuum pump in staining solution. Finally, the seedlings were incubated at 37 °C for 24 h in the dark. After staining, the seedlings were mounted in a clearing solution for observation. Images of the seedlings were taken using a stereomicroscope (Leica M165, Danaher Corporation, Wetzlar, Germany).

### 4.8. Analysis of Transiently Transformed Plants under Heat Stress

After transient infection for 48 h, the plants were exposed to 40 °C for 0 h, 1 h, and 3 h. The plants were collected for histochemical staining (DAB, NBT, and Evans blue staining), expression analysis, and H_2_O_2_ measurement. H_2_O_2_ was detected by DAB staining while O^2−^ was detected by NBT staining. The treated seedlings were immersed in DAB or NBT staining solution, respectively. The staining procedures were performed according to instructions [70]. Evans blue staining was performed according to a previously published article to detect cell damage [71]. Briefly, seedlings were vacuum infiltrated in 0.1% (*w*/*v*) Evans blue solution for 15 min. The seedlings were then immersed in absolute ethanol and heated in boiling water for 10 min to remove chlorophyll for better visualization of the staining. For H_2_O_2_ content measurement, 4 independent transformed samples were used. The experimental procedure was performed according to the manual accompanying the assay kit (micromethod) (Sangon, Shanghai, China). The raw data of the H_2_O_2_ content are shown in Table S18. 

## 5. Conclusions

In this study, we conducted a comprehensive investigation of the *L. chinense Hsf* gene family. Nineteen *LcHsf* genes were identified and categorized into three groups. Hsf gene phylogeny showed an evolutionary relationship of *LcHsfs* close to *A. thaliana* and *V. vinifera*. The expansion of the *LcHsf* family has mainly occurred through WGD or segmental duplication and suffered from purifying selection, which might have resulted in a relatively small number of *LcHsf* homologues. Four *LcHsfA* and three *LcHsfB* genes are significantly upregulated under heat treatment. Under cold-stress treatment, one *LcHsfA* gene and five *LcHsfB* genes are upregulated after a three-day duration. Drought treatment induced the expression of *LcHsfA* and *LcHsfB* family homologues at different time points. Furthermore, the transient overexpression of *LcHsfA2a* in *L. hybrid* seedlings significantly reduced H_2_O_2_ levels. These data provide essential characterization of *LcHsf* genes and have identified possible candidate genes for improving stress tolerance in *Liriodendron* plants.

## Figures and Tables

**Table 1:** Protein properties of *LcHsf* family in *L. chinense*.

Gene ID	Arabidopsis Thaliana Homologous Gene ID	Number of Amino Acids	Molecular Weight (kDa)	Theoretical pI	Subcellular Localization Prediction
*Lchi08322 (LcHsfA1a)*	*At4g17750 (AtHsfA1a)*	514	56.98	4.88	Nucleus
*Lchi01220 (LcHsfA1b)*	*At5g16820 (AtHsfA1b)*	506	53.64	5.35	Nucleus
*Lchi03447 (LcHsfA2a)*	*At2g26150 (AtHsfA2)*	382	43.13	4.83	Nucleus
*Lchi00789 (LcHsfA2b)*		378	42.70	5.22	Nucleus
*Lchi25496 (LcHsfA3)*	*At5g03720 (AtHsfA3)*	487	54.34	4.89	Nucleus
*Lchi18071 (LcHsfA4a)*	*At4g18880 (AtHsfA4a)*	447	51.04	5.56	Nucleus
*Lchi03976 (LcHsfA4b)*		444	49.19	5.79	Nucleus
*Lchi25374 (LcHsfA5)*	*At4g13980 (AtHsfA5)*	505	56.30	5.50	Nucleus
*Lchi25021 (LcHsfA7)*	*At3g51910 (AtHsfA7a)*	390	45.14	5.33	Nucleus
*Lchi19584 (LcHsfA8)*	*At1g67970 (AtHsfA8)*	388	44.35	4.75	Nucleus
*Lchi01929 (LcHsfB1a)*	*At4g36990 (AtHsfB1)*	308	34.49	5.74	Nucleus
*Lchi02207 (LcHsfB1b)*		287	32.32	7.99	Nucleus
*Lchi04992 (LcHsfB2a)*	*At5g62020 (AtHsfB2a)*	325	36.33	5.44	Nucleus
*Lchi11893 (LcHsfB2b)*	*At4g11660 (AtHsfB2b)*	310	34.53	6.63	Nucleus
*Lchi17678 (LcHsfB3)*	*At2g41690 (AtHsfB3)*	233	26.67	8.24	Nucleus
*Lchi01911 (LcHsfB4)*	*At1g46264 (AtHsfB4)*	363	40.63	8.78	Nucleus
*Lchi28762 (LcHsfB5)*	*-*	193	22.36	9.51	Nucleus
*Lchi01028 (LcHsfC1a)*	*At3g24520 (AtHsfC1)*	323	36.70	6.62	Nucleus
*Lchi08814 (LcHsfC1b)*		278	32.04	7.85	Nucleus

**Table 2:** Conserved protein domain sequences of the *L. chinense LcHsf* gene family.

Protein	DBD ^1^	OD ^2^	NLS ^3^	AHA ^4^	RD ^5^	NES ^6^
LcHsfA1a	23–115	155–206	(220) NRRITAVNKKRR	(451) DSFWEQFLSA	ND ^7^	(463) LAEQMGLL
LcHsfA1b	53–90	126–179	(193) NRRIAGVNKKRR	(425) DSFWEQFLSV	ND	(475) LTEQMGLL
LcHsfA2a	40–133	162–213	(237) RKRRLP	(351) DVEVEDLAD	ND	(370) LAEQMGFL
LcHsfA2b	33–126	155–206	(222) RKELGGVGKKRR	(341) DVEVEDLAAK	ND	(360) VLVEQMGFLGSKPSNL
LcHsfA3	36–141	208–270	(245) KRKFLK	(421) DVWGNIL	ND	(468) NDLETQLGQL
LcHsfA4a	11–103	189–208	(209) KRRLPKP	(384) DVFWEQFLTE	ND	(431) VDNLTEQMGQL
LcHsfA4b	1–83	118–169	(187) KKRRLPKP	(364) DVFWEQFLTE	ND	(413) VDHLTEQMGQL
LcHsfA5	15–81	143–194	(218) KKRRLPK	(452) DMFWEQFLTE	ND	(499) DMEQLTL
LcHsfA7	73–166	196–247	(271) KKRRR	(353) DFWDELMNE	ND	(378) LTERLGYL
LcHsfA8	12–104	140–191	(215) KKRR	(359) DVLTEQMGLL	ND	(378) LTPKDKEL
LcHsfB1a	25–117	157–214	(266) NKKKR	ND	(317) LFGV	ND
LcHsfB1b	24–116	156–204	(249) KKRAR	ND	(239) LFGV	ND
LcHsfB2a	23–115	160–208	(217) VKRFR	ND	(267) LFGV	ND
LcHsfB2b	22–114	158–201	(212) EEKPPVKRFR	ND	(256) LFGV	ND
LcHsfB3	23–116	146–189	(210) VQGEKKRKR	ND	(202) LFGV	ND
LcHsfB4	22–114	167–220	(317) LFGVPLHSKKR	ND	(317) LFGV	(347) VLRKEDLGLNL
LcHsfB5	31–120	143–184	(185) EGRSNKNGP	ND	ND	ND
LcHsfC1a	6–99	115–166	(185) LREKKRR	ND	ND	ND
LcHsfC1b	1–83	99–150	(168) KRLAEKKRR	ND	ND	ND

^1^ DBD—DNA-binding domain; ^2^ OD—oligomerization domain (HR-A/B); ^3^ NLS—nuclear localization signal; ^4^ AHA—activator motifs; ^5^ RD—repressor domain; ^6^ NES—nuclear export signal; ^7^ ND—not found.

**Table 3:** The Ka/Ks values of six gene pairs in *LcHsf*.

Gene Family	Gene Pair		Ka	Ks	Ka/Ks ^1^
*LcHsf*	*LcHsfA1a*	*LcHsfA1b*	0.2729	0.9539	0.2861
	*LcHsfA2a*	*LcHsfA2b*	0.1719	2.0985	0.0819
	*LcHsfA4a*	*LcHsfA4b*	0.1580	0.7965	0.1984
	*LcHsfB1a*	*LcHsfB1b*	0.2484	0.9938	0.2500
	*LcHsfB2a*	*LcHsfB2b*	0.1874	0.8614	0.2175
	*LcHsfC1a*	*LcHsfC1b*	0.3400	1.3707	0.2481

^1^ Ka/Ks (also known as ω) is the ratio of the number of non-synonymous substitutions (Ka) per non-synonymous site to the number of synonymous substitutions (Ks) per synonymous site in a DNA or protein sequence. It is commonly used to estimate the selective pressure acting on a gene during evolution. A Ka/Ks ratio greater than 1 suggests positive selection, indicating adaptive evolution of a protein. A ratio equal to 1 indicates neutral selection, while a ratio less than 1 suggests purifying selection, indicating that natural selection is working to eliminate deleterious mutations.

## Data Availability

The datasets are available at NCBI (PRJNA679089 and PRJNA679101).

## Supplementary Materials

The following supporting information can be downloaded at: https://www.mdpi.com/article/10.3390/ijms25052733/s1. Refs. [72,73,74,75,76] are in the Supplementary.
