# Supplementary material for: Genome-Wide Analysis of the Liriodendron chinense Hsf Gene Family under Abiotic Stress and Characterization of the LcHsfA2a Gene
Source: Int J Mol Sci. 2024 Feb 27;25(5):2733. doi: 10.3390/ijms25052733 (PMC10931991; doi:10.3390/ijms25052733)
Supplement: Supplementary file 1 [file Figure S3.pdf › Figure S3.pdf]

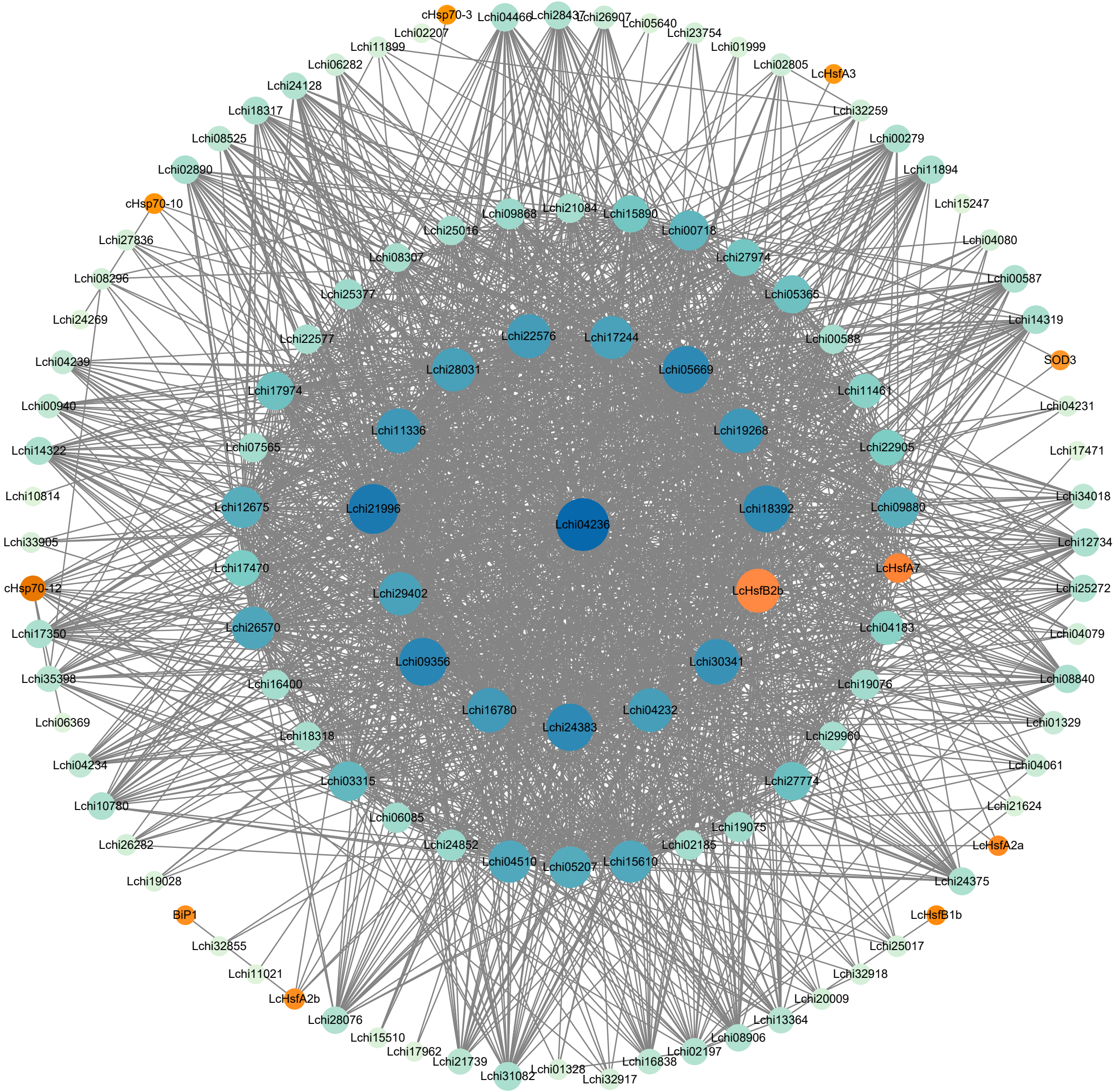

Figure S3 The correlation network of blue4 module.

Note: The network was visualized in a concentric circle diagram using Cytoscape software (v3.8.2) and was distributed using the degree algorithm. Genes with the edge weight higher than 0.60 are visualized. The LcHsf, LcHsp70 and redox genes in this figure are marked with yellow circles.

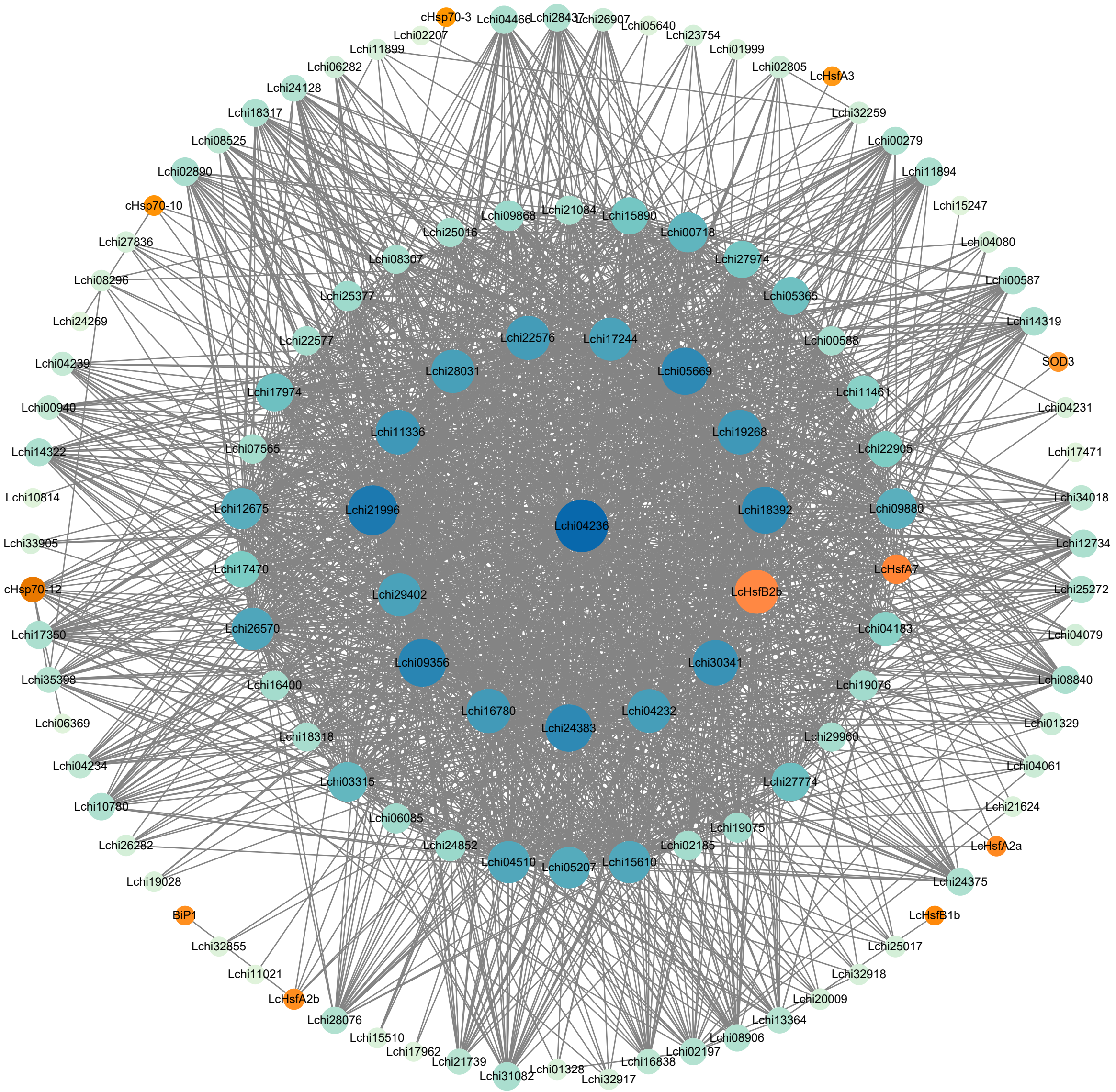

Figure S3 The correlation network of blue4 module.

Note: The network was visualized in a concentric circle diagram using Cytoscape software (v3.8.2) and was distributed using the degree algorithm. Genes with the edge weight higher than 0.60 are visualized. The LcHsf, LcHsp70 and redox genes in this figure are marked with yellow circles.
