# Supplementary material for: Genome-Wide Analysis of the Liriodendron chinense Hsf Gene Family under Abiotic Stress and Characterization of the LcHsfA2a Gene
Source: Int J Mol Sci. 2024 Feb 27;25(5):2733. doi: 10.3390/ijms25052733 (PMC10931991; doi:10.3390/ijms25052733)
Supplement: Supplementary file 1 [file ijms-25-02733-s001.zip › Figure S1.pdf]

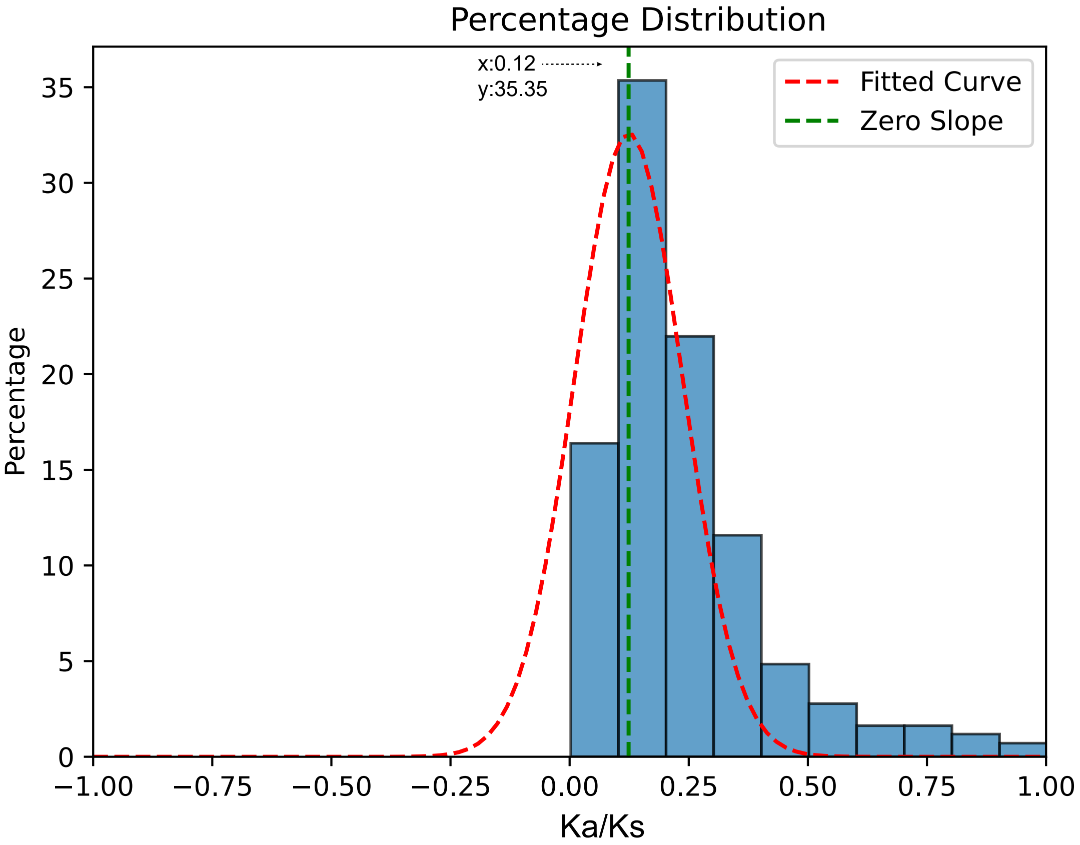

Figure S1 Percentage distribution of Ka/Ks values for all gene pairs in *L. chinense*. Note: The horizontal axis illustrates the Ka/Ks values, and the vertical axis illustrates the frequency counts for each set of items.
