# Supplementary material for: Genome-Wide Analysis of the Liriodendron chinense Hsf Gene Family under Abiotic Stress and Characterization of the LcHsfA2a Gene
Source: Int J Mol Sci. 2024 Feb 27;25(5):2733. doi: 10.3390/ijms25052733 (PMC10931991; doi:10.3390/ijms25052733)
Supplement: Supplementary file 1 [file ijms-25-02733-s001.zip › Figure S2.pdf]

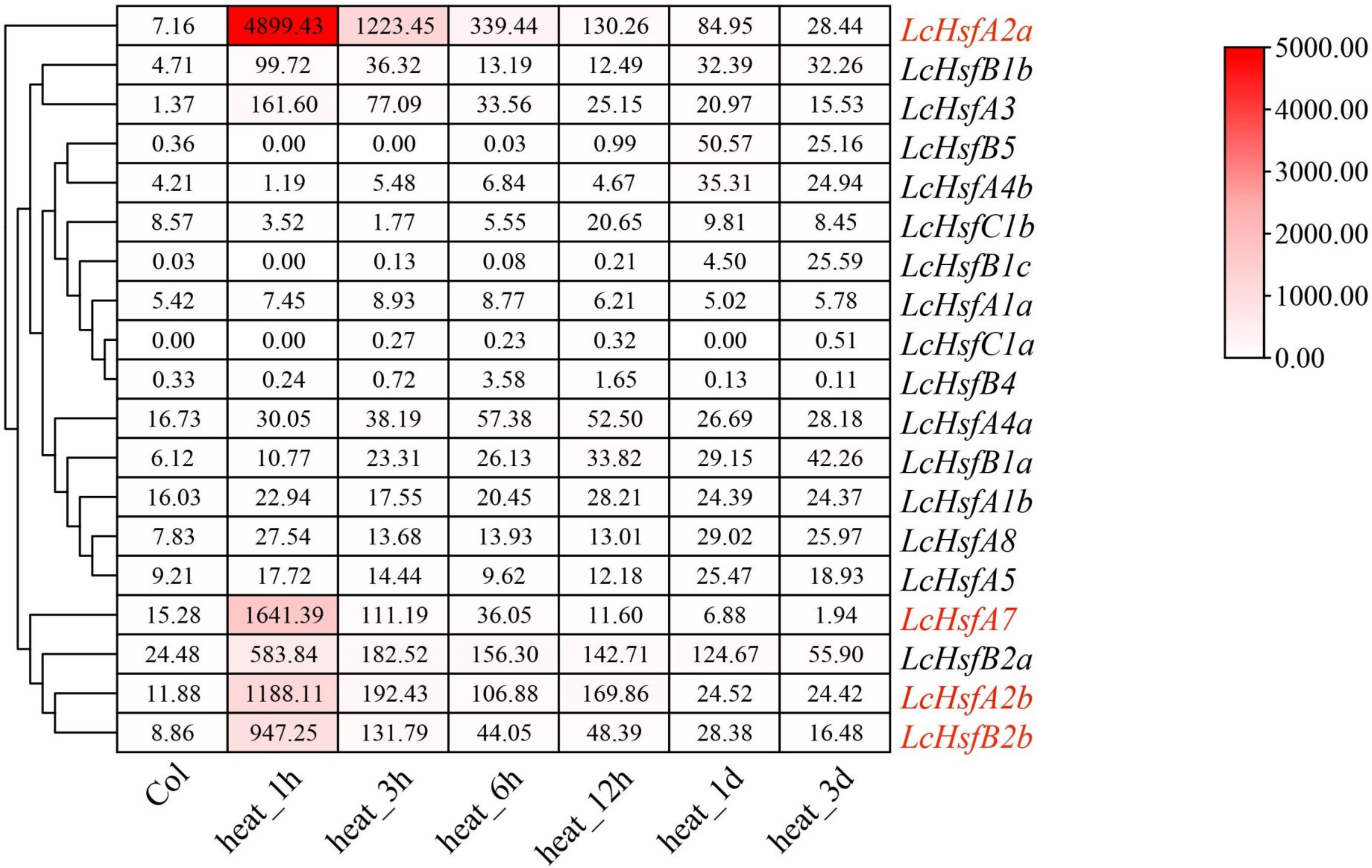

	Col	heat_1h	heat_3h	heat_6h	heat_12h	heat_1d	heat_3d
LcHsfA2a	7.16	4899.43	1223.45	339.44	130.26	84.95	28.44
LcHsfB1b	4.71	99.72	36.32	13.19	12.49	32.39	32.26
LcHsfA3	1.37	161.60	77.09	33.56	25.15	20.97	15.53
LcHsfB5	0.36	0.00	0.00	0.03	0.99	50.57	25.16
LcHsfA4b	4.21	1.19	5.48	6.84	4.67	35.31	24.94
LcHsfC1b	8.57	3.52	1.77	5.55	20.65	9.81	8.45
LcHsfB1c	0.03	0.00	0.13	0.08	0.21	4.50	25.59
LcHsfA1a	5.42	7.45	8.93	8.77	6.21	5.02	5.78
LcHsfC1a	0.00	0.00	0.27	0.23	0.32	0.00	0.51
LcHsfB4	0.33	0.24	0.72	3.58	1.65	0.13	0.11
LcHsfA4a	16.73	30.05	38.19	57.38	52.50	26.69	28.18
LcHsfB1a	6.12	10.77	23.31	26.13	33.82	29.15	42.26
LcHsfA1b	16.03	22.94	17.55	20.45	28.21	24.39	24.37
LcHsfA8	7.83	27.54	13.68	13.93	13.01	29.02	25.97
LcHsfA5	9.21	17.72	14.44	9.62	12.18	25.47	18.93
LcHsfA7	15.28	1641.39	111.19	36.05	11.60	6.88	1.94
LcHsfB2a	24.48	583.84	182.52	156.30	142.71	124.67	55.90
LcHsfA2b	11.88	1188.11	192.43	106.88	169.86	24.52	24.42
LcHsfB2b	8.86	947.25	131.79	44.05	48.39	28.38	16.48

Figure S2 Heatmap of the expression of *LcHsf* gene family members under heat stress

Note: Transcriptome FPKM values were used directly to generate heatmaps. The horizontal axis shows the heat stress time point, the vertical axis is the gene name, and the number in the figure is the FRKM value. The legend represents the magnitude of the FRKM value.
