# Supplementary material for: Genome-Wide Analysis of the Liriodendron chinense Hsf Gene Family under Abiotic Stress and Characterization of the LcHsfA2a Gene
Source: Int J Mol Sci. 2024 Feb 27;25(5):2733. doi: 10.3390/ijms25052733 (PMC10931991; doi:10.3390/ijms25052733)
Supplement: Supplementary file 1 [file ijms-25-02733-s001.zip › Figure S4.pdf]

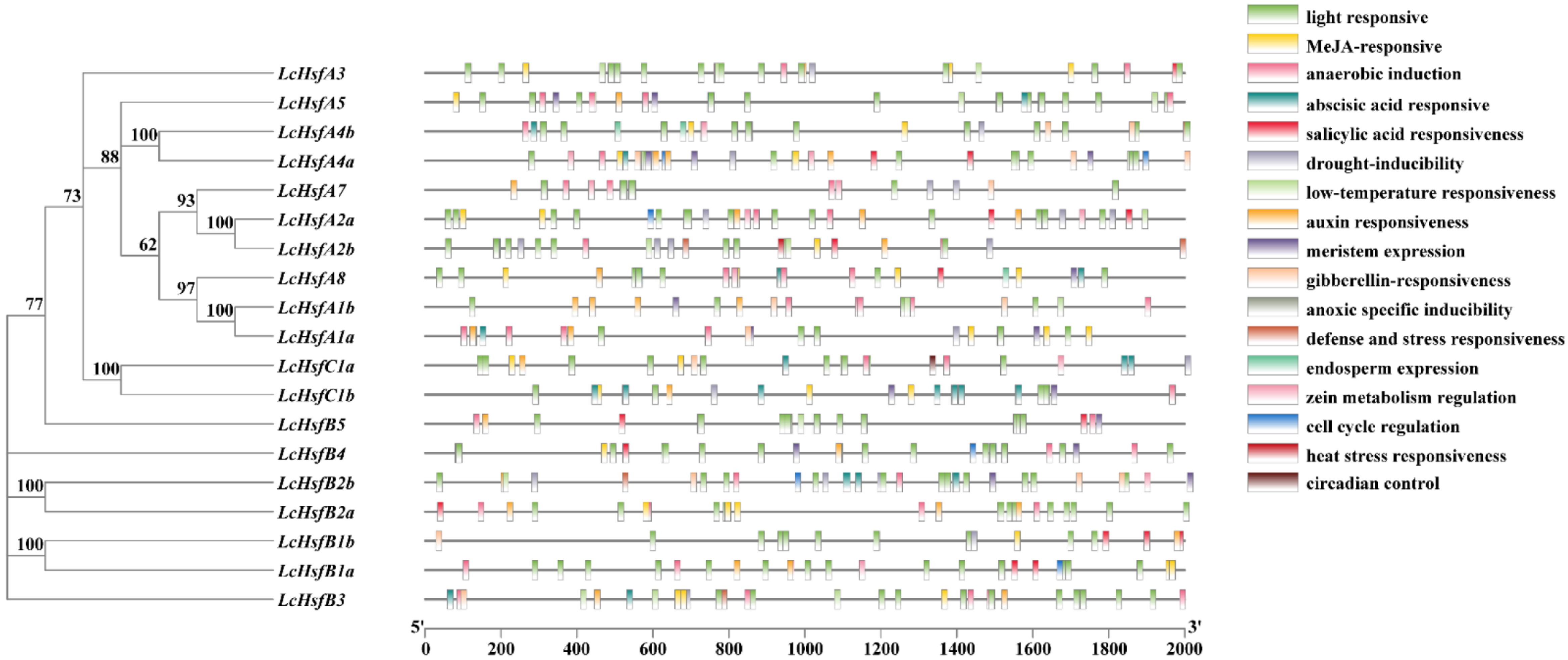

Figure S4. The *LcHsfs* genes promoter *cis*-element. Different cis-acting elements are color-coded and distributed unevenly over the sequence.
