# Supplementary material for: Genome-Wide Analysis of the Liriodendron chinense Hsf Gene Family under Abiotic Stress and Characterization of the LcHsfA2a Gene
Source: Int J Mol Sci. 2024 Feb 27;25(5):2733. doi: 10.3390/ijms25052733 (PMC10931991; doi:10.3390/ijms25052733)
Supplement: Supplementary file 1 [file ijms-25-02733-s001.zip › Figure S5.pdf]

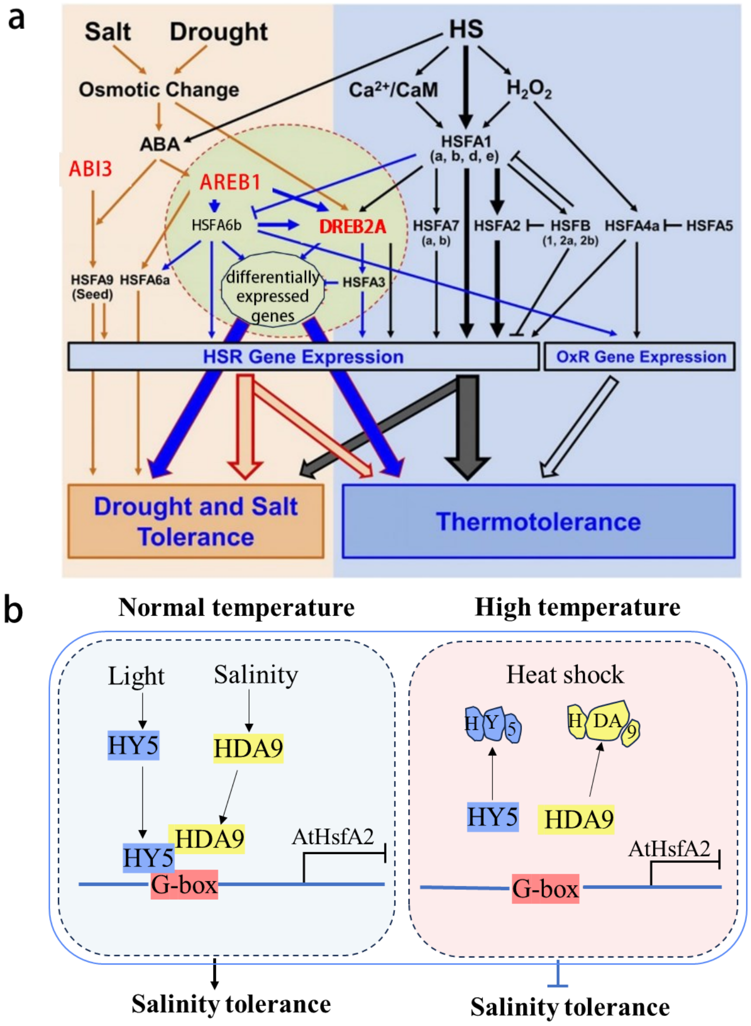

Figure S5. The TFs that bind to the *Hsf* promoter region in published reports. (a) The model of the ABA signaling pathway and ABA-mediated thermoprotection [1]. CaM: Calmodulin; OxR: oxidative stress response. (b) The schematic model shows HY5-HDA9 orchestrates the transcription of *HsfA2* to modulate salt stress response in *Arabidopsis* [2]. HDA9: histone deacetylase 9. We learned more about the transcription factors (TFs) that bind to the *Hsf* promoter region from published reports (Fig. S5a). In *Arabidopsis*, the seed-specific transcription factor ABA-insensitive 3 (ABI3) could activate the *AtHsfA9* promoter[3]. The dehydration-(DREB) family class 2 (DREB2C) interacts with two DREs located in the *AtHsfA3* promoter [4]. The promoter of *AtHsfA6a* contains two ABA-responsive elements (AREs) and is bound in vitro by three ABA-responsive TFs (AREB1, ABF3 and AREB3) [5]. The HY5 directly binds to the G-box motifs in the *AtHsfA2* promoter (Fig. S5b), to modulate the salt stress response in *Arabidopsis* [2]. With respect to heat stress, the tomato HsfA1 and HsfA2 interacted to form hetero-oligomeric superactivator complexes for synergistic activation of heat stress gene expression [6]. The interactions between *Arabidopsis* HsfA1a and HsfA1b and their interactions with HsfA2 require their oligomerization domains [7]. In conclusion, the promoter regions of *LcHsfs* genes are also rich in abscisic acid responsive elements (ABRE elements), drought inducible

elements (MBS elements), light responsive elements (G-box and Box4). Thus, the above-mentioned TFs can be used as a reference source to study the binding of *LcHsf* genes promoter, such as ABA-responsive TFs (AREB1, ABF3 and AREB3) or TF HY5.

References

1. Huang, Y. C.; Niu, C. Y.; Yang, C. R.; Jinn, T. L., The Heat Stress Factor HSFA6b Connects ABA Signaling and ABA-Mediated Heat Responses. *Plant physiology* **2016,** 172, (2), 1182-1199.
2. Yang, J.; Qu, X.; Li, T.; Gao, Y.; Du, H.; Zheng, L.; Ji, M.; Zhang, P.; Zhang, Y.; Hu, J.; Liu, L.; Lu, Z.; Yang, Z.; Zhang, H.; Yang, J.; Jiao, Y.; Zheng, X., HY5-HDA9 orchestrates the transcription of HsfA2 to modulate salt stress response in Arabidopsis. *Journal of Integrative Plant Biology* **2023,** 65, (1), 45-63.
3. Kotak, S.; Vierling, E.; Bäumlein, H.; von Koskull-Döring, P., A novel transcriptional cascade regulating expression of heat stress proteins during seed development of Arabidopsis. *Plant Cell* **2007,** 19, (1), 182-95.
4. Chen, H.; Hwang, J. E.; Lim, C. J.; Kim, D. Y.; Lee, S. Y.; Lim, C. O., Arabidopsis DREB2C functions as a transcriptional activator of HsfA3 during the heat stress response. *Biochem Biophys Res Commun* **2010,** 401, (2), 238-44.
5. Hwang, S. M.; Kim, D. W.; Woo, M. S.; Jeong, H. S.; Son, Y. S.; Akhter, S.; Choi, G. J.; Bahk, J. D., Functional characterization of Arabidopsis HsfA6a as a heat-shock transcription factor under high salinity and dehydration conditions. *Plant, cell & environment* **2014,** 37, (5), 1202-22.
6. Chan-Schaminet, K. Y.; Baniwal, S. K.; Bublak, D.; Nover, L.; Scharf, K. D., Specific interaction between tomato HsfA1 and HsfA2 creates hetero-oligomeric superactivator complexes for synergistic activation of heat stress gene expression. *The Journal of biological chemistry* **2009,** 284, (31), 20848-57.
7. Li, M.; Berendzen, K. W.; Schöffl, F., Promoter specificity and interactions between early and late Arabidopsis heat shock factors. *Plant Mol Biol* **2010,** 73, (4-5), 559-67.
